# Height difference between the vestibular and palatal walls and palatal width: a cone beam computed tomography approach

**DOI:** 10.1186/s12903-020-01322-0

**Published:** 2021-03-15

**Authors:** P. López-Jarana, C. M. Díaz-Castro, A. Falcão, C. Falcão, J. V. Ríos-Santos, A. Fernández-Palacín, M. Herrero-Climent

**Affiliations:** 1 Porto Dental Institute, Porto, Portugal; 2 Madrid Perio-Implantes, Madrid, Spain; 3 Faculty of Dentistry Oporto University (FMDUP), Porto, Portugal; 4 Health Sciences Faculty, Fernando Pessoa University, Porto, Portugal; 5 School of Dentistry, Universidad de Sevilla, C/ Avicena S/N, 41009 Seville, Spain; 6 Dpto. Ciencias Sociosanitarias, Universidad de Sevilla, Seville, Spain

**Keywords:** Palatal bone wall thickness, Buccal and palatal bone wall height, Cone beam computed tomography (CBCT)

## Abstract

**Background:**

The objective of this study was to measure two parameters involved in tri-dimensional implant planning: the position of the buccal and palatal bone wall and the palatal thickness.

**Methods:**

Cone beam computed tomography (CBCT) images (Planmeca ProMax 3D) of 403 teeth (208 upper teeth and 195 lower teeth) were obtained from 49 patients referred to the Dental School of Seville from January to December 2014. The height difference between the palatal and buccal walls was measured on the most coronal point of both walls. The thickness of the palatal wall was measured 2 mm from the most coronal point of the palatal wall.

**Results:**

The mean values in the maxilla were 1.7 ± 0.9 mm for central and lateral incisors, 2.2 ± 1.7 mm for canines, 1.6 ± 0.9 mm for premolars and 1.9 ± 1.5 mm for molars. In the lower jaw, the mean values were 1.3 ± 0.8 mm for incisors, 1.7 ± 1.2 mm for canines, 2.3 ± 1.3 mm for premolars, and 2.6 ± 1.7 mm for molars. In the upper jaw, more than 55% of maxillary teeth (excluding second premolars and molars) presented mean height differences greater than 1 mm. In the mandible, more than 60% of incisors showed a buccal bone thickness of 1 mm from the apical to lingual aspect. All teeth except the second premolar presented a buccal wall located more than 1 mm more apically than the lingual bone wall.

**Conclusions:**

The buccal bone wall is located more apically (greater than 1 mm) than the palatal or lingual table in most of the cases assessed. The thickness of the palatal or lingual table is also less than 2 mm in the maxilla and mandible, except in the upper canines and premolars and the lower molars.

## Background

Recently, publications about the cone beam computed tomography diagnostic test have increased in impact journals, and have been used to assess the anatomy of several anatomical accidents [1, 2]. An elementary principle in surgical procedures is knowledge of the anatomy of the area. When a non-maintainable tooth must be removed, it can be replaced with a dental implant. This replacement can be performed at different times after tooth extraction. Immediate implants are those that are placed on the patient immediately after tooth extraction, which is described inside the different implant loading protocols of Hämmerle et al. [3]. In 2017, Buser et al. [4] recommended that the clinician should check the patient for a series of previous anatomical parameters before moving towards an immediate implant protocol. These parameters are the thickness, height and integrity of the buccal bone wall, the height and thickness of the palatal bone wall, the width of the mesial and distal ridge of the postextraction alveolus, the height and inclination of the dentoalveolar process, etc.

The height of the palatal bone wall and the palatal thickness are less studied parameters. The buccal bone wall position has been considered a referential point to take into consideration at the three-dimensional dental implant position [5]. This buccal bone wall in its most coronal portion is usually formed by a thin layer of bone that limits and is intimately related to the periodontal ligament; this bone is also called the fasciculate or bundle bone. The bone bundle disappears after the extraction, which results in a greater reduction of the vertical height of the vestibular wall of the alveolus, although there is also reabsorption of the lingual wall [6]. The height of the vestibular wall of a thin cortex is remodelled to a greater extent than that of a thick cortex. According to Rossi et al. [7], the lingual alveolar bone exhibits a medium bone resorption of 0.6 mm, 0.7 mm and 0.5 mm at 1, 3 and 5 mm apical to the crest after immediate implant installation. Knowledge of the mean width parameters of the palatal bone plate would help the clinician determine the posterior behaviour of the palatal bone plate after immediate implant insertion. The palatal bone wall serves as an anatomic source to achieve primary stability for the immediate implant protocol [8]. The existence of an alveolar palatal bone alveolus will allow, in the case of indicating an immediate implant, achievement of the necessary stability of the implant, promoting proper healing [9]. The objectives of this study were as follows: first, to describe the position of the most coronal portion of the vestibular cortex with respect to the most coronal portion of the lingual or palatal cortical in the apex-coronal direction in the different locations. The second objective was to examine the thickness of the palatal table 2 mm from the crest.

## Methods

### Study type

The present transversal descriptive study included CBCT images (Planmeca ProMax 3D PLANMECA OY Asentajankatu 6 FIN-00880 Helsinki) from patients referred to the Dental School of Seville from January to December 2014. A total of 49 patients (mean age of 40.3 years) satisfied the inclusion criteria (19 men and 30 women), resulting in a sample of 407 teeth analyzed. Four teeth were declared void for statistical reasons, and thus, 403 teeth were finally evaluated (208 upper teeth and 195 lower teeth). CBCTs using a spiral technique with 0.2 mm thickness (voxel size 200 µm, 307 × 200 mm field of view (FOV), 90 kV, 10 mAs, 1 mm pass). The ethics committee of the University of Seville approved this non-interventional study for the acquisition of the images, number 0159-N-14 (PEIBA) of the Junta de Andalucía, Spain. The inclusion criteria were as follows: absence of systemic disease or previous bad health (particularly ruling out bone diseases, uncontrolled or poorly controlled diabetes, unstable or life-threatening conditions or requirement for antibiotic prophylaxis); absence of radiotherapy or chemotherapy in the past 5 years; autoimmune diseases and any drug use; pregnancy. The local exclusion criteria were a radiolucent image comprising greater than 1/3 of the root; severe root angulation or root resorption; and radiographic evidence of guided bone/tissue regeneration.

The CBCTs used for the present study were selected from the faculty’s database; the images were not specifically acquired for this study. The CBCTs were anonymous and saved inside a protected computer with specific software for implant planning. The images were saved in Digital Imaging and Communications in Medicine (DICOM) format and measured with Adobe Photoshop and with implant software using 3 precalibrated examiners. The captured images of the scan were saved with the standard zoom of the Planmeca Romexis viewer and exported to Photoshop CS5 to be measured. The scans used for the study were performed on a Planmeca ProMax 3D RX device that uses a spiral technique with a thickness of 0.2 mm of cut (200 µm as the size of the voxel, 307 × 200 mm of field of vision (FOV), 90 kV, 10 mAs and 1 mm pitch).

Three examiners were dental specialists in the field of periodontal and implant dentistry and were calibrated in order to join the measurement criteria. The calibration was achieved by blind measurements of the same random teeth by the three examiners, registering the grade of reproducibility of ten radiograph. The intra-examiner intraclass correlation coefficient (ICCs) were 0.98, 0.97 and 0.98, and the inter-examiner ICCs were 0.99 and 0.98.

**Fig. 1:**
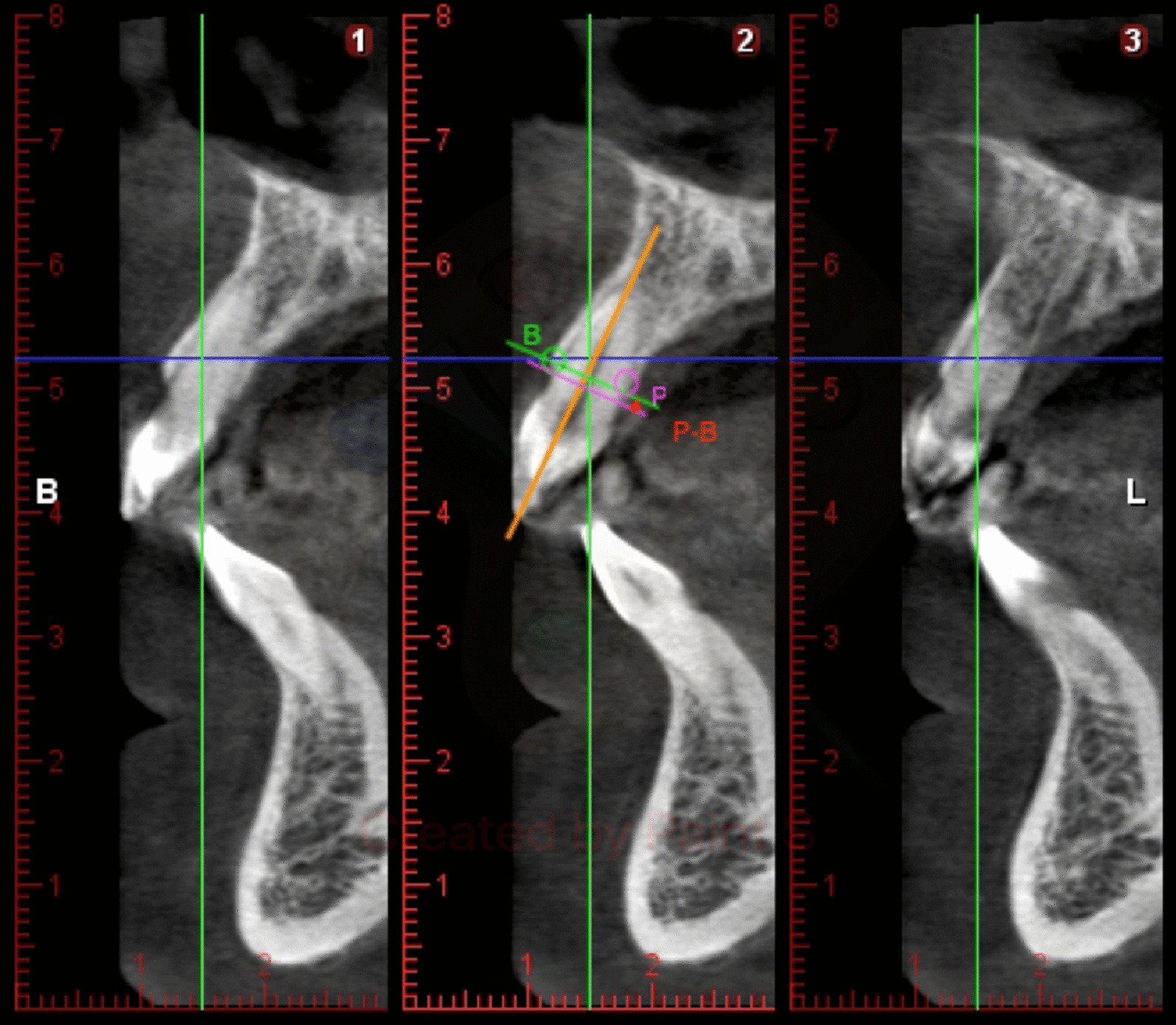
Height difference between the buccal and palatal wall. The distance between the perpendicular line and the palatal ridge is measured, drawing a line that is in turn parallel to the axial axis of the tooth, up to the point where the palatal crest begins

The radiographic images of the CBCTs were analysed on two computers, with both Windows 7 and Intel core i-7 processors with a monitor resolution of 1366 × 768. Data were reconstructed with an image size of 401 × 401 × 401, voxel size of 200 µm, 90 kV, 14 mA, 12.249 s and DAP 12.3 (mGyxcm^2^). The thickness of the alveolar bone was evaluated by viewing the cross-sectional image made at the centre of the tooth parallel to its long axis (Fig. 1). To perform the measurements, sagittal scans from the reconstructed data were selected, resulting in images where the entire root and cement-enamel junction (CEJ) were present for single rooted teeth. Several different slices were selected for multirooted teeth, one for each root. The captured scan images were saved with the standard zoom of Planmeca Romexis viewer and exported to Photoshop CS5 to be measured. The captured images had a resolution of 72 pixels/inch and were saved with the standard zoom of Planmeca Romexis viewer and exported to Photoshop CS5 to be measured. The measurements were performed using commercial image analysis and graphics software Adobe Photoshop CS5, Adobe Systems Incorporated, 345 Park Avenue, San Jose, California 95110, USA. The slice image exported to Adobe Photoshop CS5 had a rule that brought the opportunity to calibrate the measured image made with the photo editor to the DICOM image.

### Variables

#### Difference in the height of the palatal wall with respect to the vestibular wall

The axial axis of the tooth is drawn, and a perpendicular line passes through the vestibular crest at the coronal vestibular point. The distance between the perpendicular line and the palatal ridge is measured, drawing a line that is in turn parallel to the axial axis of the tooth up to the point where the palatal crest begins. Positive values will be given if the palatal ridge is located more coronally than the vestibular cortex, and negative values will be given if the palatal or lingual bone crest is more apical than the vestibular crest (Fig. 1).

#### Palatal thickness

**Fig. 2:**
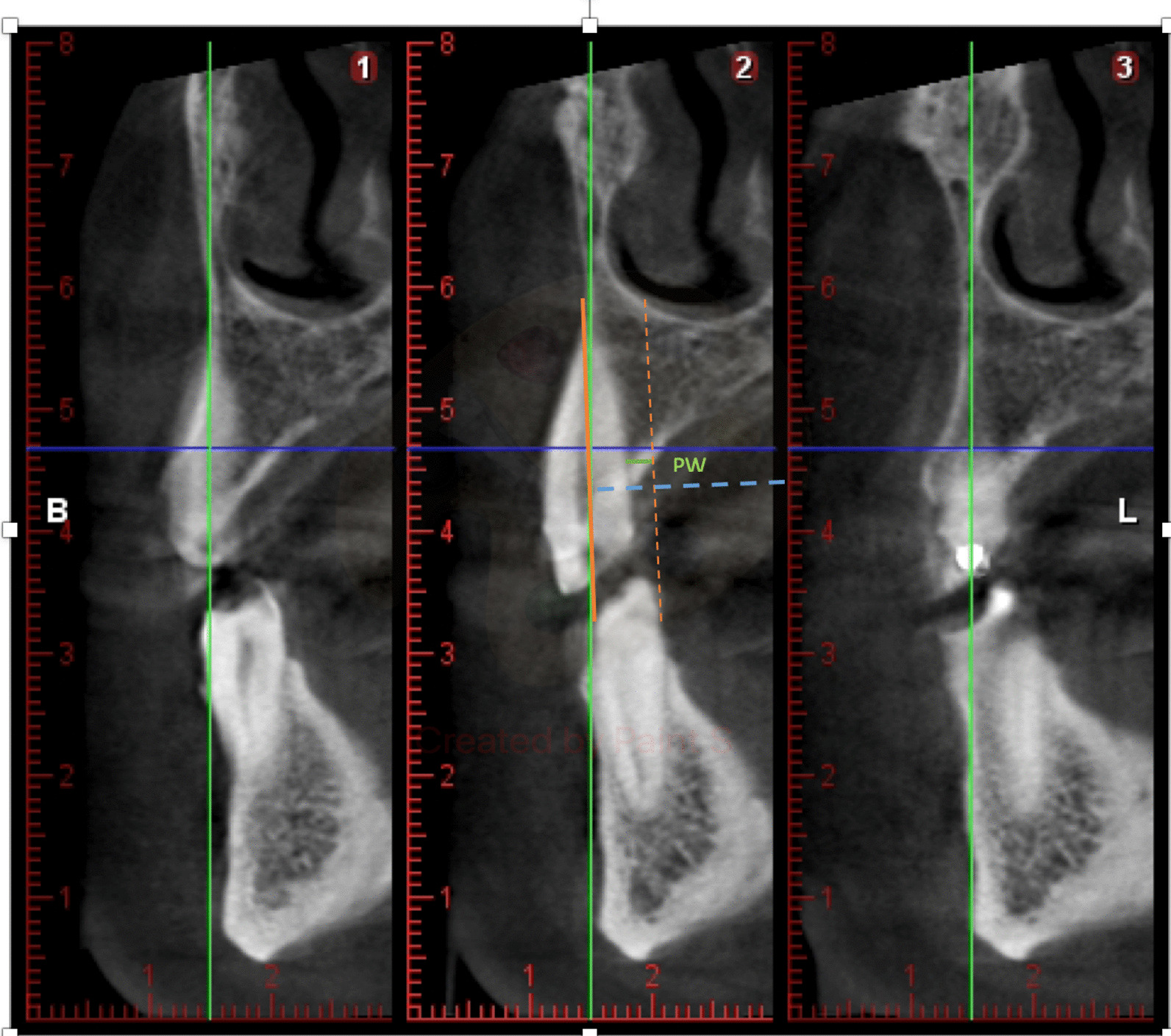
Palatal thickness. Distance measured from the root surface to the outer surface of the palatal table at the most coronal height or closest to the CEJ thickness

Measurement of the thickness or thickness of the crest, in the palatal-palatal direction, perpendicular to the line that marks the axial axis of the tooth at the most coronal point of the palatal table. Distance measured from the root surface to the outer surface of the palatal table at the most coronal height or closest to the CEJ in the palatal aspect (Fig. 2).

### Statistical analysis

The data obtained were introduced in Excel software (Microsoft) to perform a descriptive analysis with adequate codification of the patients. The data were analysed using SPSS software version 22. Descriptive statistics, including the mean, SD, and 95% confidence intervals (ICs), were calculated.

## Results

The mean values of palatal thickness over all dental groups. The mean values for the maxilla were 1.7 ± 0.9 mm for central and lateral incisors, 2.2 ± 1.7 mm for canines, 1.6 ± 0.9 mm for premolars and 1.9 ± 1.5 mm for molars. At the mandible, the mean values were 1.3 ± 0.8 mm for incisors, 1.7 ± 1.2 mm for canines, 2.3 ± 1.3 mm for premolars, and 2.6 ± 1.7 mm for molars. From the mandible, the median values for the central and lateral incisors were 1.3 ± 0.8 mm; for mandibular canines, 1.7 ± 1.2 mm; for mandibular premolars, 2.3 ± 1.3 mm; and for mandibular molars, 2.6 ± 1.7 mm. The mean values of palatal thickness were lower than 2 mm for all upper teeth except for the canines. At the mandible, the molars and premolars presented greater values of residual palatal bone thickness. For the upper jaw, canines and premolars showed higher mean values; nevertheless, we had to take into consideration that these locations also presented lower values. At the mandible, the sample tendency seemed to be the same, but lower values were found at the incisors and premolars.

The second variable for all dental groups, the palatal wall, seemed to be 1 mm more coronal than the buccal bone wall, as follows: On the upper jaw: 19 of 32 central incisors, 19 of 34 lateral incisors, 22 of 33 canines, 18 of 25 first premolars, 8 of 22 s premolars, 24 of 36 first molars and 11 of 23 s molars. Greater values were found in the anterior aesthetic zona and molars.

**Fig. 3:**
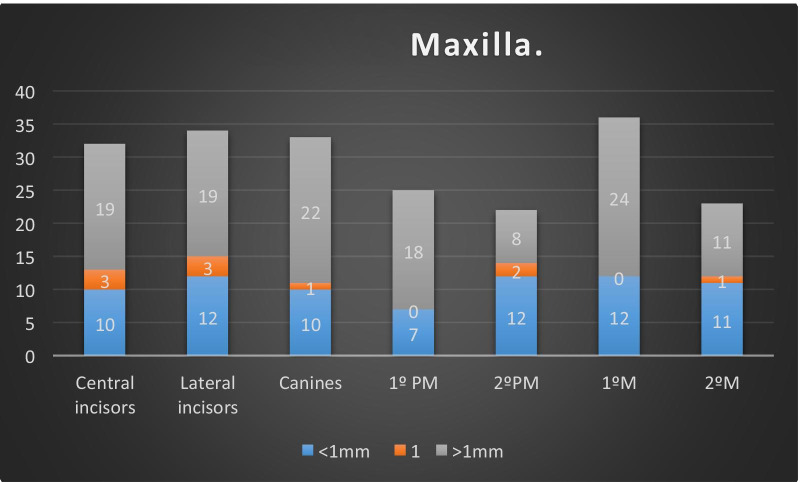
Height difference between the buccal and palatal wall

**Fig. 4:**
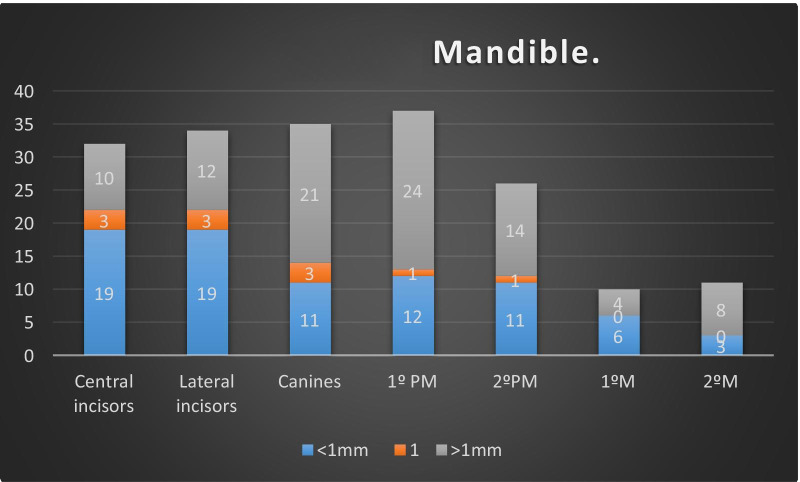
Difference height between buccal and lingual

It was found that 59.4% of the central incisors, 55.8% of the lateral incisors, 66.6% of the canines, 72% of the first premolars, 36.3% of the second premolars, 66.7% of the first molars and 47.8% of the second molars presented mean values of a greater than 1 mm height difference (Fig. 3). For the mandible, the sample showed a different behaviour. For the incisor, the buccal bone wall was registered more coronally than the lingual bone wall. Taking this into consideration, 19 of 32 (59.4%) central incisors and 19 of 34 (55.8%) lateral incisors showed a buccal bone thickness of 1 mm from apical to lingual. For 21 of the 35 (60%) canines, the buccal bone was located more than 1 mm apically than the lingual bone wall. The measurements of the rest of the sample showed this result for 24 of 35 (68.6%) first premolars, 14 of 26 (54%) second premolars, 4 of 10 (40%) first molars and 8 of 11 (72.2%) second molars (Fig. 4).

## Discussion

The long-term aesthetic implications of bone remodelling after implant placement are focused on the apical migration of the gingival margin, a parameter associated with the disappearance of the bundle bone. Therefore, the need for knowledge of the dimensions around the teeth is highlighted in order to predict the reabsorption that may occur after the extraction of a tooth and its replacement by means of an implant [10].

The morphology of the underlying bone around a tooth before an immediate implant plays a fundamental role in soft tissue stability and influences the long-term aesthetic result of the final restoration [11]. There are many factors responsible for the possible aesthetic risks simultaneous to the placement of the immediate implants: absence of the bundle bone, remodelling of the alveolar bone after tooth extraction, thickness of the buccal bone wall and convexity of the alveolar process. All these parameters are related to the emerging profile of the final restoration [12]. The present study helps to elucidate the socket bone morphology, especially for immediate implant protocols. Several recent studies have focused on the thickness of the buccal bone wall [13–15]. The palatal wall represents an anatomical part of the socket that could provide bone anchorage for implant installation. In terms of important anatomical parameters to take into consideration in implant planning, the apical position of the immediate implant is a crucial clinical decision for stable aesthetic results. From our research, the doctor has to consider that the palatal bone wall can induce a surgical error because of its position. Therefore, especially on immediate implant, its apical-coronal position should be more submerged on the palatal aspect to avoid a possible posterior vestibular exposition of the implant neck. Furthermore, the palatal bone thickness helps the clinician choose the proper indication for immediate implantation to reach primary stability. Another clinically important aspect could be related to prosthodontic procedures. After immediate implant healing, the tri-dimensional position of the implant neck at the palatal aspect could require bone around the neck or abutment remodeling to construct the final crown restoration. CBCT can be used to study the thickness of alveolar bone. The determination of the degree of reproducibility and the precision of the values obtained by this type of radiological test have been quantified in several studies on cadavers [16]. The measurements of the bone tissue obtained with the CBCT were accurate and differed scarcely from the physical findings. These differences, which are between − 0.13 and + 0.13 mm, are not statistically significant [17]. The measurements that are obtained depend on the software used, according to several studies, although the variability does not usually exceed 1 mm [18]. In this study, the software used did not allow the measurement of thicknesses less than 0.8 mm for safety reasons, so Adobe Photoshop CS5 software was used to measure all thicknesses (including those considered minimum) by subsequently converting them to the real values. The mean values for the molars of our study are similar to those in other publications, and our results show that 55% of the first molars and 40% of the second molars have a thickness of the vestibular table less than 1 mm, as also found in the study of Matsuda et al. [19] In that study, healthy first molars were evaluated, and the researchers found that the thickness of the palatal table at a 2 mm height of the crest was 1.34 ± 0.54 mm. Our thickness values are similar to those of the palatal bone wall, with a mean value of 1.9 ± 1.5 mm [19]. In the research published by Yoshimine et al. [20], 10 linear parameters and 1 angular measurement in 30 locations of 30 patients using CBCT were evaluated. The horizontal thickness of the palatal values was 1.74 ± 073 mm in the first premolars and 1.76 ± 0.76 mm in the second premolars. For the mesial root of the first molars, the means of palatal thickness were 1.58 ± 1.23 mm and 1.71 ± 1.1 mm in the distal root. For the second molars, the mesial root was 2.43 ± 1.18 mm in the mesial root and 2.47 ± 1.28 mm in the distal root [20]. In the study of AlTarawneh et al. [21], measurements were made on CBCT of teeth in the anterior aesthetic sector. There were a total of 120 patients in whom the vestibular and palatal tables were measured in the coronal, middle and apical thirds. The thickness of the palatal table was significantly greater. For the central incisor, the median value was 1.6 mm; for the lateral incisor, it was 1.5 mm; and for the canines, it was 1.7 mm [21]. Their results are similar to ours; only the mean values for the canines present greater numbers. In our study, we included the posterior sectors of the maxilla and mandible that represent locations where immediate implants or postextraction can be placed. These locations have not been as studied as the previous sectors at the anatomical level by using CBCT.

## Conclusions

Within the limitations of a study of these characteristics, such as the sample size both in terms of the patients studied and the locations analysed, we can conclude that for the analysed sample corresponding to a population of the characteristics described above, the buccal bone wall is located more apically (greater than 1 mm) than the palatal or lingual table in most of the cases assessed. In addition, the thickness of the palatal or lingual table is also less than 2 mm in the maxilla and mandible, except in the upper canines and premolars and lower molars. Studies of these characteristics are necessary to understand the anatomical characteristics and therefore their limitations, especially when clinicians want to choose treatments such as immediate implants.

## Data Availability

Data are available at the following link: https://idus.us.es/xmlui/handle/11441/79894 (Research Repository of the University of Seville) (open).

## Abbreviations

CBCT: Cone beam computed tomography
CEJ: Cementoenamel junction
CIs: Confidence intervals
DICOM: Digital imaging and communications in medicine
FOV: Field of view
ICC: Intraclass correlation coefficient
PEIBA: Portal de Ética de la Investigación Biomédica de Andalucía (Spain

## References

[1] Duruel O, Ataman-Duruel ET, Tozum MD, Karabulut E, Tozum TF (2019). The radiological evaluation of posterior superior alveolar artery topography by using computed tomography. Clin Implant Dent Relat Res.

[2] Tassoker M (2020). What are the risk factors for maxillary sinus pathologies?. A CBCT study Oral Radiol.

[3] Hammerle CH, Chen ST, Wilson TGJ (2004). Consensus statements and recommended clinical procedures regarding the placement of implants in extraction sockets. Int J Oral Maxillofac Implants.

[4] Buser D, Chappuis V, Belser UC, Chen S (2000). Implant placement post extraction in esthetic single tooth sites: when immediate, when early, when late?. Periodontology.

[5] Levine RA, Ganeles J, Gonzaga L, Kan JK, Randel H, Evans CD (2017). 10 keys for successful esthetic-zone single immediate implants. Compend Contin Educ Dent.

[6] Araujo MG, Sukekava F, Wennstrom JL, Lindhe J (2005). Ridge alterations following implant placement in fresh extraction sockets: an experimental study in the dog. J Clin Periodontol.

[7] Rossi F, Romanelli P, Ricci E, Marchetti C, Botticelli D (2013). A cone beam tomographic evaluation of hard tissue alterations at immediate implants: a clinical prospective study. Int J Periodontics Restorative Dent.

[8] Cardaropoli G, Araujo M, Lindhe J (2003). Dynamics of bone tissue formation in tooth extraction sites. An experimental study in dogs. J Clin Periodontol.

[9] Elian N, Cho SC, Froum S, Smith RB, Tarnow DP (2007). A simplified socket classification and repair technique. Pract Proced Aesthet Dent.

[10] Vera C, De Kok IJ, Chen W, Reside G, Tyndall D, Cooper LF (2012). Evaluation of post-implant buccal bone resorption using cone beam computed tomography: a clinical pilot study. Int J Oral Maxillofac Implants.

[11] (International team for oral implantology) ITI consensus conference. Vitznau, Switzerland, 1997. Proceedings. Clin Oral Implants Res. 2000;11 Suppl 1:1–158. 11447967

[12] Roe P, Kan JY, Rungcharassaeng K, Caruso JM, Zimmerman G, Mesquida J (2012). Horizontal and vertical dimensional changes of peri-implant facial bone following immediate placement and provisionalization of maxillary anterior single implants: a 1-year cone beam computed tomography study. Int J Oral Maxillofac Implants.

[13] Huynh-Ba G, Pjetursson BE, Sanz M, Cecchinato D, Ferrus J, Lindhe J (2010). Analysis of the socket bone wall dimensions in the upper maxilla in relation to immediate implant placement. Clin Oral Implants Res.

[14] Januario AL, Barriviera M, Duarte WR (2008). Soft tissue cone-beam computed tomography: a novel method for the measurement of gingival tissue and the dimensions of the dentogingival unit. J Esthet Restor Dent..

[15] Kim YJ, Park JM, Kim S, Koo KT, Seol YJ, Lee YM (2016). New method of assessing the relationship between buccal bone thickness and gingival thickness. J Periodontal Implant Sci.

[16] Patcas R, Muller L, Ullrich O, Peltomaki T (2012). Accuracy of cone-beam computed tomography at different resolutions assessed on the bony covering of the mandibular anterior teeth. Am J Orthod Dentofacial Orthop.

[17] Pauwels R, Araki K, Siewerdsen JH, Thongvigitmanee SS (2015). Technical aspects of dental CBCT: state of the art. Dentomaxillofac Radiol.

[18] Gonzalez-Martin O, Oteo C, Ortega R, Alandez J, Sanz M, Veltri M (2016). Evaluation of peri-implant buccal bone by computed tomography: an experimental study. Clin Oral Implants Res.

[19] Matsuda H, Borzabadi-Farahani A, Le BT (2016). Three-dimensional alveolar bone anatomy of the maxillary first molars: a cone-beam computed tomography study with implications for immediate implant placement. Implant Dent.

[20] Yoshimine S, Nishihara K, Nozoe E, Yoshimine M, Nakamura N (2012). Topographic analysis of maxillary premolars and molars and maxillary sinus using cone beam computed tomography. Implant Dent.

[21] AlTarawneh S, AlHadidi A, Hamdan AA, Shaqman M, Habib E (2018). Assessment of bone dimensions in the anterior maxilla: a cone beam computed tomography study. J Prosthodont.

